# Snowflake Bionic Flow Channel Design to Optimize the Pressure Drop and Flow Uniform of Proton Exchange Membrane Fuel Cells

**DOI:** 10.3390/mi13050665

**Published:** 2022-04-24

**Authors:** Yuting Li, Jingliang Bi, Miao Tang, Gui Lu

**Affiliations:** 1 School of Energy Power and Mechanical Engineering, North China Electric Power University, Beijing 102206, China; Liyt0914@163.com (Y.L.); t117807749@gmail.com (M.T.); 2 CNNC Key Laboratory on Nuclear Reactor Thermal Hydraulics Technology, Nuclear Power Institute of China, Chengdu 610213, China; bijingliang0126@163.com

**Keywords:** PEM fuel cell, flow channel, bionic flow field, multi-objective genetic algorithm optimization

## Abstract

The flow channel design of bipolar plates plays a significant role in the proton exchange membrane fuel cells operation, particularly in thermal and water management. The pursuit of low-pressure drop supply and flow field uniformity in PEM fuel cells has not stopped, resulting in numerous new bipolar plate flow channel designs. The biomimetic leaf vein shape-based flow channel and lung flow channel designs can significantly improve gas supply uniformity and reduce pressure drop. Therefore, we propose a snowflake-shaped bionic channel design by integrating the advantages of the leaf vein shape and lung shape channel. A 3D multi-physics fuel cell model is used to verify the feasibility and superiority of the bionic snowflake design in improving fuel cell performance, especially in reducing the pumping work. The local pressure distribution, oxygen distribution, water distribution, and current density distribution are used to reveal the enhancement mechanism of the new snowflake flow channel. The flow uniformity is further enhanced by using multi-objective (13 target parameters) and multi-parameter (18 independent variables) genetic algorithm optimization. The general goal of this work is to provide a new strategy for the thermal and water management of PEM fuel cells.

## 1. Introduction

Proton exchange membrane (PEM) fuel cells are electrochemical devices that convert hydrogen energy into electricity [1,2]. PEM fuel cells have become a promising substitute for fossil-fueled technologies because of their high efficiency, low operating temperature, fast start-up, low noise, and low emissions [3,4]. With such advantages, PEM cells have great potential applications in the automotive industry, small stationary power generation systems, or distributed energy systems [5,6]. 

The bipolar plates play significant roles in the PEM fuel cells, such as distributing fuel and oxidant, collecting and transporting current, removing product gas and liquid water, and providing structural rigidity for the cell stack assembly. Hence, the bipolar plate design has a significant impact on the performance of the fuel cell, which has been studied extensively [3,4]. The commonly used microchannel styles include parallel straight channel, serpentine, cross-finger [5], dotted [6], and so forth. These channel styles are simple and easy to fabricate, but there is still much room for improvement and optimization in the aspects of reactant uniformity, pressure drop, and water removal [7,8]. To deal with such issues, various channel designs have been proposed, such as fractal channels [9], composite flow channels [10], spiral flow channels [11], radial flow channels [12,13], and optimized pin-type flow channels [14]. These novel types of flow channels aim to make the reactants uniformly distributed inside the cell and facilitate water removal, thus improving the overall performance of the fuel cell.

In addition to these novel flow channels, a series of bionic structural flow channel designs inspired by nature are also proposed, such as leaf veins [15,16,17,18,19,20], lung tubes [21,22,23,24], and lymphatic topology [25]. These novel bionic designs aim to solve the issues of flow field uniformity [16], gas diffusion and liquid water removal [17], and system pressure drop [21]. Table 1 compares current flow channel designs in PEMFCs. In summary, the general principles of the flow channel design are to address the following issues: (1) according to Murray’s law, the size of the channel should be proportional to its volumetric flow requirements; (2) when using a branching structure, the branching angle should be less than 90-degree bends to avoid stagnation zones and the short cut of flows; (3) the flow field design should take the reactant consumption along the flow channel into account; (4) the appropriate proportion of the flow channel area and rib area in order to provide sufficient oxygen and effective conductivity of the electrode plate over the cathode reaction zone; (5) subcostal convection can facilitate the oxygen transport in gas diffusion layer; and (6) provide effective liquid water removal inside the cathode to prevent clogging. 

According to these principles, neither a single leaf vein design nor a single lung tube design will suffice. For example, the lung design is favorable for the small pressure drop, while the leaf vein branching design is better for the flow uniformity. However, both pressure drop and uniformity are favorable for the flow channel design of fuel cells. Therefore, we introduce a new flow channel design inspired by nature’s snowflake, which considers both the lung fractal and leaf vein branching bionic structures. Compared with the traditional flow channel designs, the new flow channel design can achieve better gas diffusion and liquid water removal performance with lower pressure drops. Fuel gas mass transport, microchannel liquid water management, overall pressure drop, and fuel cell performance are examined by a 3D multiscale and multi-physical model. The flow channels are further optimized by a multi-objective optimization method based on a genetic algorithm. The general goal of this work is to provide a new strategy for the thermal and water management of PEM fuel cells.

## 2. Numerical Model

### 2.1. Geometric Model

**Figure 1:**
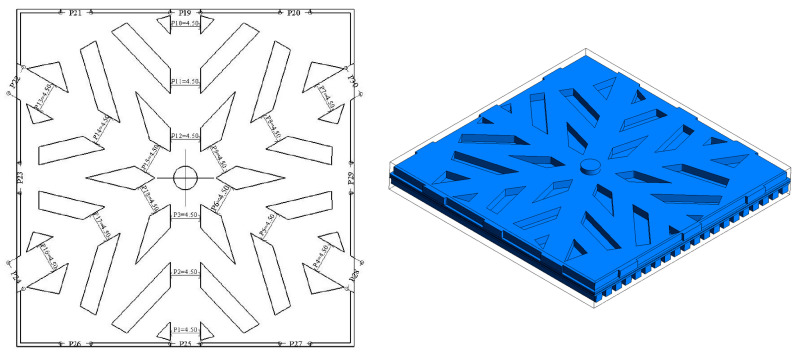
Snowflake bionic flow channel design.

A PEM fuel cell model with a 3D sandwich structure is built in this work, which has been widely used to study mass transport and energy conversion numerically. The PEM fuel cell comprises five parts: bipolar plates, flow channels, gas diffusion layer, catalytic layer, and proton exchange membrane. Since the cathode flow channel is more significant for the PEM fuel cell than the anode, we only induce the snowflake design for the cathode flow channel. The details of the design geometry are shown in Figure 1. The width of the six main channels, branch, surrounding channel, and outlet of the snowflake bionic flow channel are 4.5 mm, 3.5 mm, 0.75 mm, and 0.5 mm. The circular inlet is located in the center of the bipolar plate with a diameter of 3.5 mm. The total area is 25 cm^−2^, with a channel area ratio of 58.29%. The thicknesses of the proton exchange membrane, catalyst layer, gas diffusion layer, and collector plate are 0.15 mm, 0.01 mm, 0.35 mm, and 2.5 mm, respectively. The height of the anode and cathode channel is 1.5 mm. 

### 2.2. Governing Equations

Different positions in the fuel cell correspond to different governing equations, which are divided into two parts: fluid flow and electrochemical reaction. Three-dimensional, single-phase, isothermal PEM models include momentum equations, continuity equations, conservation equations, water content equations, and potential equations. The following assumptions are induced to build the multi-physical model. The fluid is non-Newtonian, incompressible, flowing micro-laminar flow, and constant physical properties. The porous layers are all isotropic.

The continuity equation is solved by

(1) $$\nabla \cdot (\rho \vec{u})=S_{m}$$

The momentum equation is solved by

(2) $$\frac{1}{\varepsilon^{2}}\nabla \cdot (\rho \vec{u}\vec{u})=-\nabla P+\frac{1}{\varepsilon }\nabla (\mu \nabla \vec{u})+S_{\vec{u}}$$

The source term $S_{\vec{u}}$ is the resistance source term of the porous structure

(3) $$S_{\vec{u}}=-\frac{\mu \vec{u}}{k_{P}k_{\mathrm{rg}}}-\frac{\varepsilon C_{F}\rho }{\sqrt{k_{P}}}\left|\vec{u}\right|\vec{u}$$

The component equation is solved by

(4) $$\nabla \cdot (\rho \vec{u}C_{k})=\nabla \cdot (\rho D_{k,eff}\nabla C_{k})+S_{m}$$

The source term $S_{m}$ is solved by

(5) $$S_{m}=S_{H_{2}}$$

(6) $$S_{m}=S_{H_{2}O}+S_{O_{2}}$$

(7) $$S_{H_{2}}=-(j_{a}/2F)M_{H_{2}}$$

(8) $$S_{O_{2}}=-(j_{c}/4F)M_{O_{2}}$$

(9) $$S_{H_{2}O}=(j_{c}/2F)M_{H_{2}O}$$

The property, such as two-phase density, is calculated by,

(10) $$\rho_{mix}=\frac{P_{op}}{RT\sum \left(\frac{X_{k}}{M_{k}}\right)}$$

The transfer equation of product water in the flow channel, gas diffusion layer, and catalytic layer is calculated by,

(11) $$\nabla \cdot (\frac{\rho_{l}k_{p}k_{rl}}{\mu_{l}}\frac{\partial P_{c}}{\partial s}\nabla s)-\nabla \cdot (\frac{\rho_{l}k_{p}k_{rl}}{\mu_{l}}\nabla P)+\nabla \cdot (\frac{n_{d}M_{H_{2}O}}{F}\vec{i_{m}})=0$$

The product water transfer equation in the membrane is calculated by

(12) $$\nabla \cdot ((\frac{\alpha_{d}M_{H_{2}O}}{F}\vec{i_{m}})\lambda -(\frac{M_{H_{2}O}\rho_{dry}}{M_{m}}D_{\lambda })\nabla \lambda )=0$$

The electrochemical reaction proceeds in the catalytic layer and the reaction equation is

(13) $$\nabla \cdot ((\frac{\alpha_{d}M_{H_{2}O}}{F}\vec{i_{m}})\lambda -(\frac{M_{H_{2}O}\rho_{dry}}{M_{m}}D_{\lambda })\nabla \lambda )=0$$

The current collector and membrane potentials are calculated as:

(14) $$\nabla \cdot (\sigma_{S}\nabla \Phi_{S})=-S_{j}$$

(15) $$\nabla \cdot (\sigma_{m}\nabla \Phi_{m})=S_{j}$$

The source term $S_{j}$ is solved by

(16) $$S_{j}=-j_{a}=Aj_{0,a}^{ref}(\frac{C_{H_{2}}}{C_{H_{2}}^{ref}})\left[e^{(\alpha_{a}F/RT)\eta }-\frac{1}{e^{(\alpha_{c}F/RT)\eta }}\right]$$

(17) $$S_{j}=j_{c}=Aj_{0,c}^{ref}(\frac{C_{O_{2}}}{C_{O_{2}}^{ref}})\left[e^{(\alpha_{a}F/RT)\eta }-\frac{1}{e^{(\alpha_{c}F/RT)\eta }}\right]$$

Assuming that the anode potential is equal to the ground potential,

(18) $$\Phi_{s}^{c}-\Phi_{s}^{a}=V_{cell}\Rightarrow \Phi_{s}^{c}-0=V_{cell}\Rightarrow V_{cell}=\Phi_{s}^{c}$$

**Figure 2:**
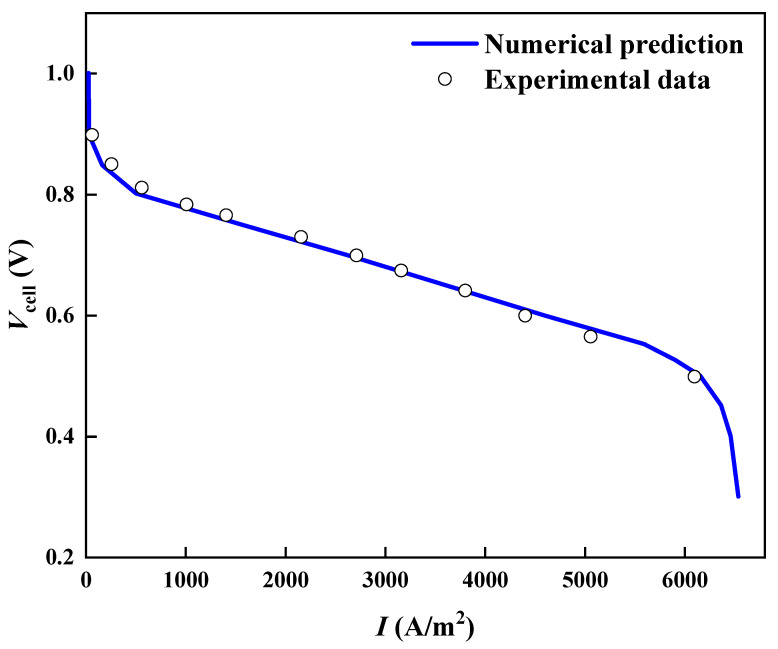
Model validation.

The initial temperature of the PEMFC for this study is 353 K. The anode inlet fuel is 20% H_2_O, 80% H_2_, which has a mass flow rate of 2.7 × 10^−7^ kg s^−1^. The cathode inlet is 10% H_2_O, 20% O_2_, and 70% N_2_, with a mass flow rate of 7.5 × 10.6 kg s^−1^. The physical property parameters are shown in Table 2. The model validation is shown in Figure 2, which shows a good agreement between the experiments and the present multi-physical model. 

## 3. Results and Discussion

### 3.1. Feasibility and Advantage of Snowflake Bionic Flow Channel

**Figure 3:**
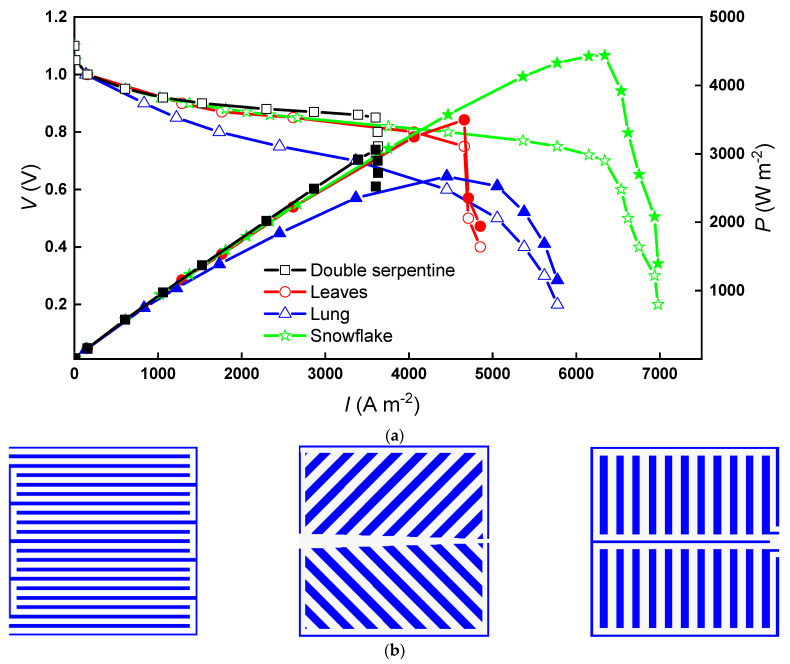
The polarization curve and power density curve of PEM fuel cells with various designs: (**a**) U-I curve; (**b**) dual serpentine, leaf, and lung designs.

Figure 3 shows the polarization curve and the power density curve for the snowflake bionic flow channel with three classical flow channels, dual serpentine (DS), leaf (LE), and lung (LU). At high potentials, the difference in cell current density between the leaf-shaped flow channel, snowflake flow channel, and double-snake flow channel designs is small, but all three flows are higher than the lung-shaped flow channel. When the potential is between 0.92 V and 0.82 V, the current density of the PEM fuel cell with the dual serpentine flow channel is slightly higher than that of the lung flow channel and the snowflake flow channel, with a maximum difference of 1060 A m^−2^. This region is in the ohmic polarization region, in which the main reason affecting the current output is the need to overcome the resistance of electron conduction through the electrode material and various connecting components and the ion conduction through the electrolyte. Therefore, the resistance of the leaf-shaped flow channel, lung-shaped flow channel, and snowflake flow channel cells are all greater than that of the dual serpentine flow field cell.

**Figure 4:**
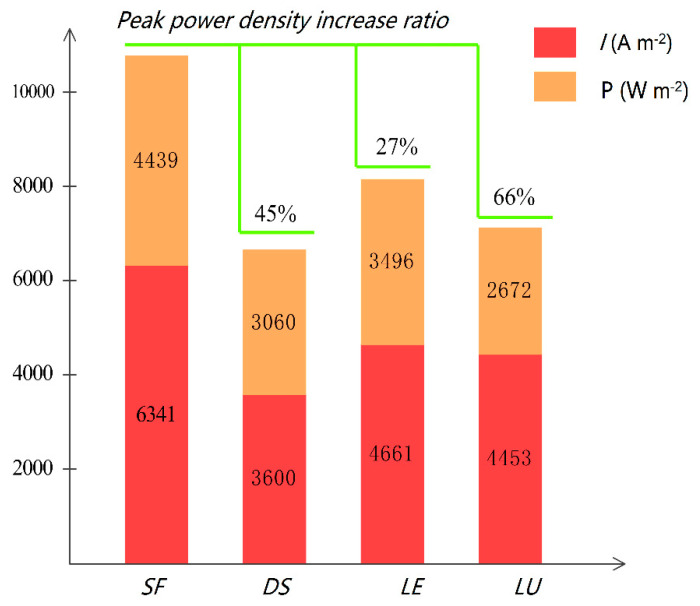
Peak power density of PEM fuel cells with various flow channels.

As the potential decreases, the current density in the dual serpentine flow channel hardly changes and enters the concentration polarization region early. The current of the PEM fuel cell with the leaf-shaped flow channel, lung-shaped flow channel, and snowflake flow channel will continue to increase. The current density corresponding to the occurrence of concentration polarization in the snowflake channel is 1.7 times higher than that in the dual serpentine channel fuel cell, 1.3 times higher than that in the leaf-shaped, and 1.4 times higher than that in the lung-shaped channel fuel cell. When the leaf flow channel, snowflake flow channel, and dual serpentine flow channel enter into the concentration difference polarization zone, the current density of the lung-shaped flow channel still increases with the decrease of potential. The overall polarization curve is relatively flat. The unique concentration polarization characteristics of the lung-shaped flow channel are closely related to its water management, which will be discussed in detail below. The concentration of reactant gases in the concentration polarization zone is heavily depleted, resulting in a low concentration of reactants on the electrode surface. Due to mass transfer limitations, sufficient reactants cannot be supplied to the electrode surface, resulting in voltage loss. Therefore, it can be expected that the snowflake flow channel has better mass transfer performance in the reaction interface. Figure 4 shows the peak power density values for the various flow channels. A maximum of 66% increases the snowflake-shaped flow channel’s peak power density compared to the other three flow channels.

The performance-enhancing mechanism of the snowflake flow channel can be explored by the local transfer characteristics of cathode pressure drop, oxygen mass fraction, water content and current density in the membrane.

### 3.2. Local Distribution and Transport Characteristics for the Snowflake Flow Channel

The overall pressure drop in the fuel cell flow channel is critical for evaluating fuel cell performance, which is relevant to the pump power to feed the fuel and oxidant to the fuel cell [30,31]. More minor pressure loss indicates a higher cost-benefit for cell power generation. In contrast, a large pressure drop can force more fluid fuel or oxidant to penetrate the diffusion layer to the catalytic layer for reaction. At the same time, it will enhance the forced convection of liquid water and facilitate the removal of product water. Therefore, finding an optimal pressure drop value is significant to improving the fuel cell performance.

Table 3 compares the pressure drop of the four flow channels inside the cathode bipolar plate. Pressure drop is calculated as the area-weighted average pressure at the inlet minus the area-weighted average pressure at the outlet. The snowflake-shaped flow channel designed in this paper has a vertical inflow in the middle and outflow all around. Hence, the overall pressure drop is tiny, with an average pressure drop of only about 1.1 Pa under ideal conditions. The average pressure drop in the double serpentine flow channel is 56.40 Pa, the average pressure drop in the leaf-shaped flow channel is 8.48 Pa, and the average pressure drop in the lung-shaped flow channel is 7.92 Pa. As the reaction gas is gradually consumed from the inlet to the outlet, the local pressure in the flow channel gradually decreases from the inlet to the outlet. The slightest pressure drop in the snowflake-shaped flow channel makes the reactant gas diffuse thoroughly and uniformly throughout the flow channel, increases the concentration of reactants on the catalytic layer surface, and delays the concentration polarization of the whole cell. The slight pressure drop allows the snowflake flow channel to not cause local fuel supply shortage due to a too fast local reaction, which significantly affects the cell performance.

**Figure 5:**
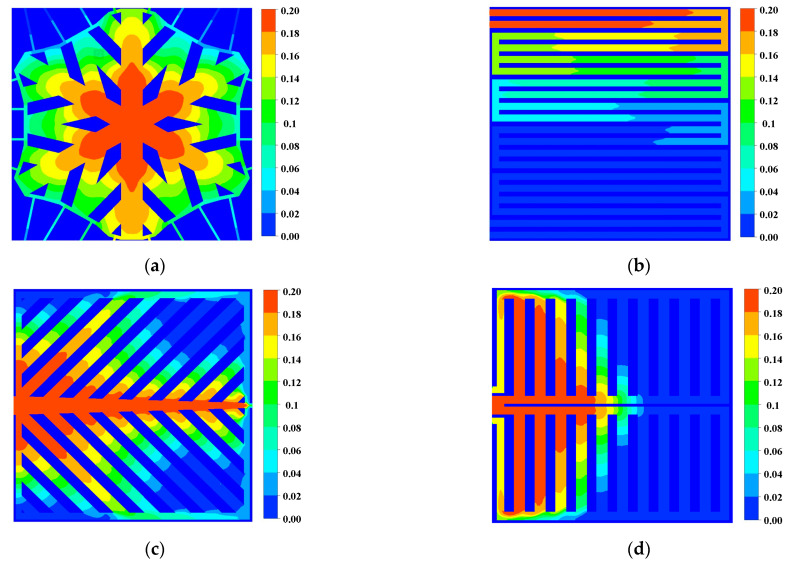
Oxygen mass fraction in the flow channel (0.8 V): (**a**) Snowflake bionic flow channel; (**b**) dual serpentine flow channel; (**c**) leaf-shape bionic flow channel; (**d**) lung-shape bionic flow channel.

**Figure 6:**
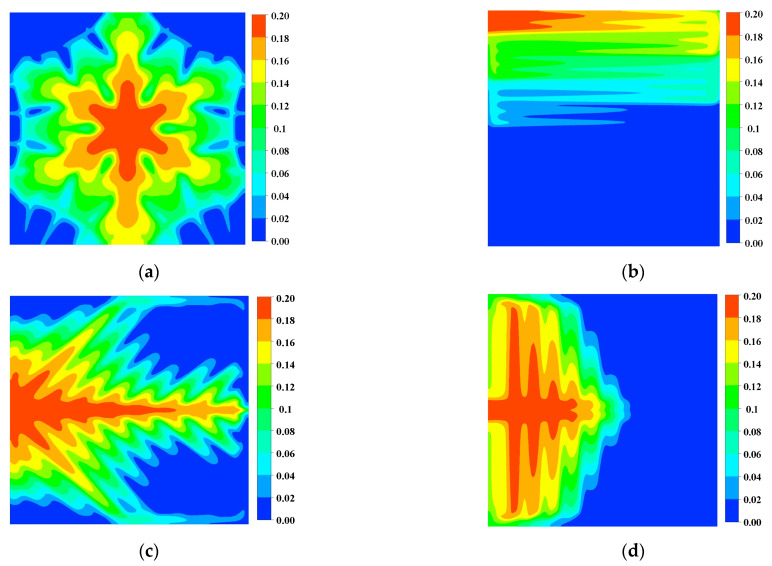
Oxygen mass fraction of cathode gas diffusion layer (0.8 V): (**a**) Snowflake bionic flow channel; (**b**) dual serpentine flow channel; (**c**) leaf-shape bionic flow channel; (**d**) lung-shape bionic flow channel.

The oxygen content directly affects the electrochemical reaction rate of the PEM fuel cell. As shown in Figure 5, the oxygen mass fraction in the second half of the snowflake flow channel is reduced to 0.06. The local mass fractions in the other three channels are almost 0, indicating that the cell experiences a local gas supply deficiency, which affects the overall cell performance. In addition, the distribution of oxygen in the flow channel region of the snowflake flow channel is more uniform than that of the other flow channels. Comparing the area of the high concentration region in Figure 6 with Figure 5, it can be found that the oxygen content in the diffusion layer will be slightly lower than that in the channel. With sufficient oxygen supply in the snowflake flow channel, the leaf-shaped flow channel, and the lung-shaped flow channel, there is no fuel outflow at the exit of the dual serpentine flow channel and the oxygen supply is insufficient. Therefore, the fuel utilization rate of snowflake, leaf, and lung channels is lower than that of double serpentine channels. The lack of local fuel supply in the leaf-shaped and lung-shaped channels is mainly due to poor water management. The snowflake channel is mainly due to low channel resistance and rapid oxygen flow through the cell channel. Thus, the channel topology should be further optimized, which will be discussed in the following section.

**Figure 7:**
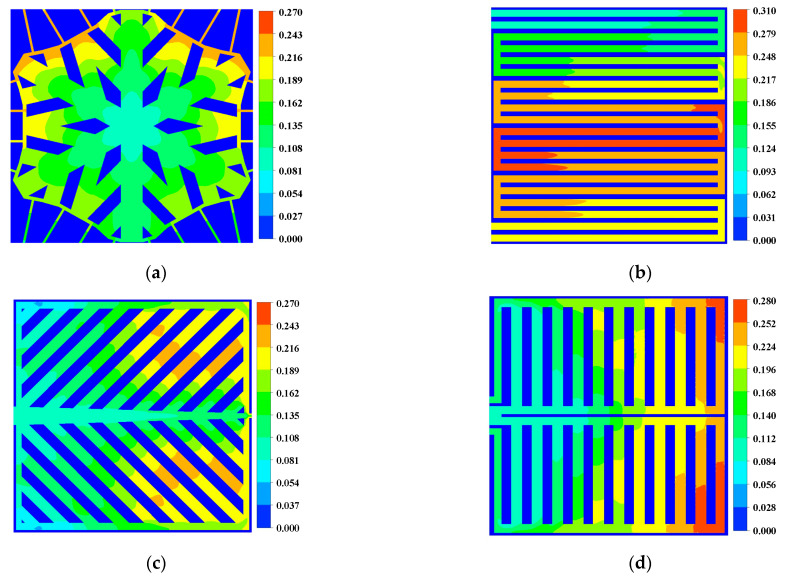
Water mass fraction in cathode channel (0.8 V): (**a**) Snowflake bionic flow channel; (**b**) dual serpentine flow channel; (**c**) leaf-shape bionic flow channel; (**d**) lung-shape bionic flow channel.

**Figure 8:**
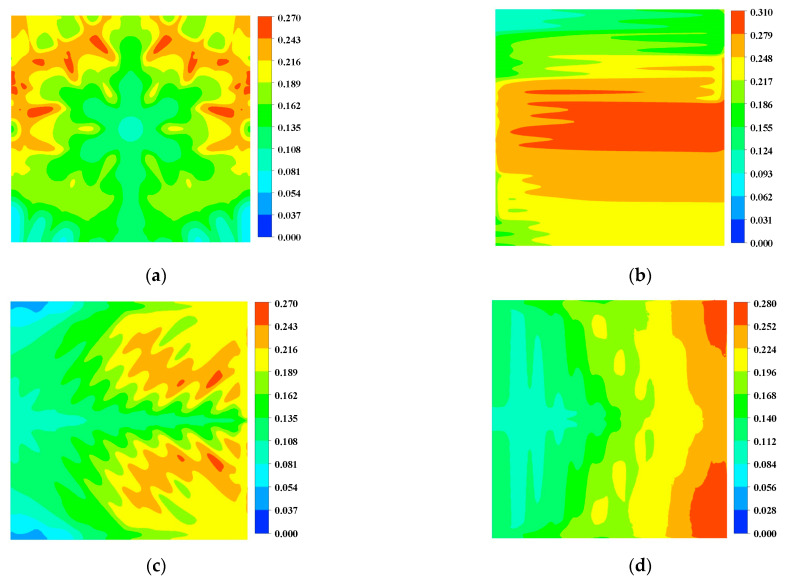
Water mass fraction in cathode diffusion layer (0.8 V): (**a**) Snowflake bionic flow channel; (**b**) dual serpentine flow channel; (**c**) leaf-shape bionic flow channel; (**d**) lung-shape bionic flow channel.

Figure 7 and Figure 8 show water distribution within the flow channel and diffusion layer. In the middle region of the dual serpentine flow channel, the liquid water content, the maximum is 31% and the leaf-shaped and lung-shaped flow channels have the most liquid water at 27% and 28% downstream of the flow channel. In the snowflake flow channel, the liquid water concentration is near the outlet, which has much less effect on the electrochemical reaction than the double serpentine flow channel, the leaf-shaped flow channel, and the lung-shaped flow channel. In addition, the maximum value of water content in the snowflake channel is also lower than that in the dual serpentine channel and the lung-type flow channel. The distribution of liquid water within the diffusion layer was similar. Therefore, the water management of the snowflake flow channel bionic structure flow channel is better than other comparison flow channels.

**Figure 9:**
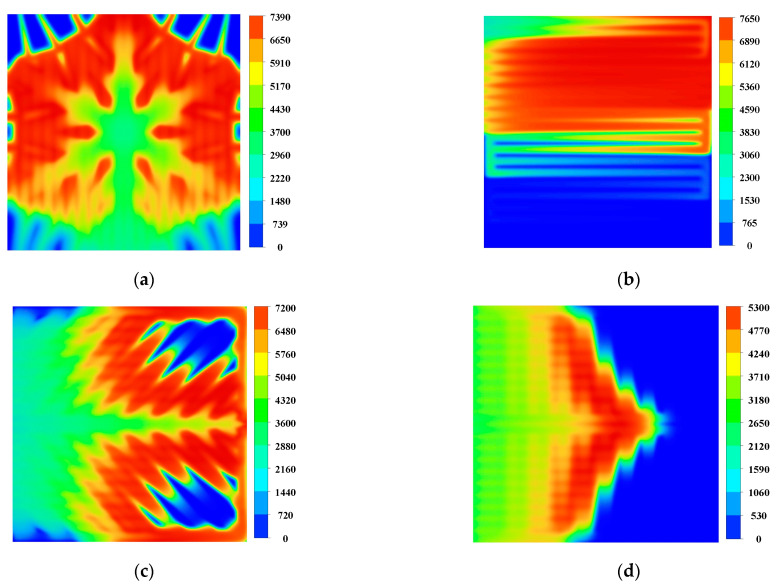
The current density distribution of the membrane (A m^−2^) (0.8 V): (**a**) Snowflake bionic flow channel; (**b**) dual serpentine flow channel; (**c**) leaf-shape bionic flow channel; (**d**) lung-shape bionic flow channel.

The current density of the fuel cell can directly reflect the electrochemical reaction of the cell, which can specifically reflect the cell’s performance locally and provide guidance to the improving design of the fuel cell. Figure 9 shows the current density distribution in the membrane. The gas flow rate is the largest in the inlet region, so that the electrochemical reaction does not have time to occur. In this region, the current density generated will be smaller than other parts. The current density in the second half of the flow channel is almost zero, with a surface average current density of 3641.78 A m^−2^. The oxygen supply in the double serpentine flow channel is insufficient. At the same time, there is a right angle at the turn, where a current surge occurs. The leaf-shaped and lung-shaped channels also have local fuel supply deficiencies because of poor water management, with face-averaged current densities of 4099.66 A m^−2^ and 1980.22 A m^−2^. On the other hand, the snowflake-shaped channel is relatively homogeneous, with no right angles present in the primary reaction area and a face-averaged current density of 4656.20 A m^−2^, which is 135.14% higher than that of the lung-shaped channel.

### 3.3. Muti-Objection and Multi-Parameter Optimization of the Snowflake Flow Channel

**Figure 10:**
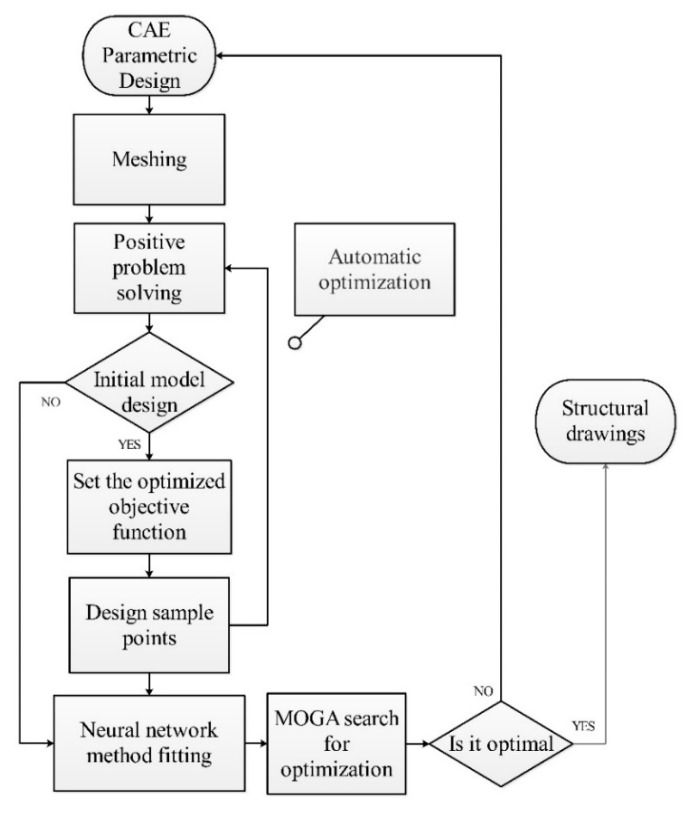
Flow diagram of MOGA optimization for the snowflake flow channel fuel cells.

**Figure 11:**
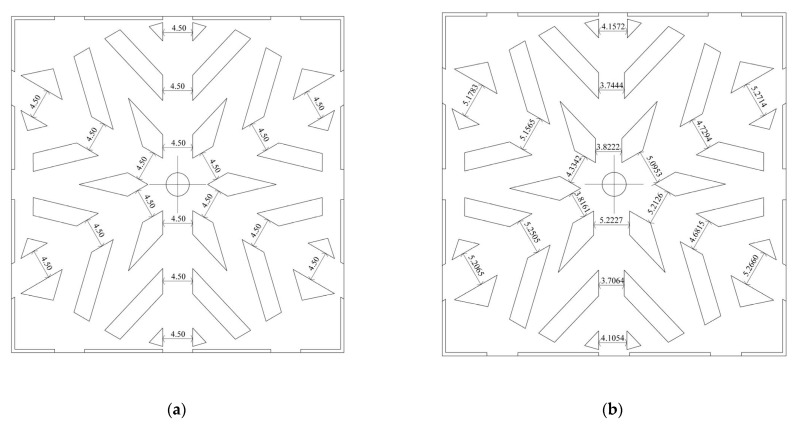
The MOGA optimization for snowflake flow channel fuel cell: (**a**) initial design parameters; (**b**) optimized parameters.

In summary, the snowflake flow channel has proven effective for oxygen diffusion and water removal with less pressure drop punished. However, we can see the limitation of this novel flow channel from the local transport characteristics. For example, the pressure drop of the snowflake flow channel is so tiny that the oxygen flows through the fuel cell too quickly to be consumed. Secondly, the area ratio of the channel is too small. The ribs around the flow channel block the gas flow and diffuse to the diffusion layer. The existence of the capillary outlet will hinder the transportation of water to a certain extent. Such limitations can be improved by using topology optimization. To do this, the parametric design was implemented to search the optimization value of flow channel topology automatically, as shown in Figure 10. There are 18 designed parameters in the snowflake channels shown in Figure 11, which are the input parameters of the optimization. The selected input parameter is the rib spacing of the six main roads, the mass flow of each exit, and the degree of deviation between them, as shown in Table 4 and Figure 11. The optimized objective is the uniform mass flow rates of 12 cathode outlets. To quantize this effect, a new uniform parameter, which is the differences among these flow rates of 12 outlets, is defined as,

(19) P31 = (P19 − P20) × 2 + (P19 − P21) × 2 + (P23 − P22) × 2 + (P23 − P24) × 2 +  (P25 − P26) × 2 + (P25 − P27) × 2 + (P29 − P28) × 2 + (P29 − P30) × 2

## 4. Conclusions

In this work, we propose a snowflake-shaped bionic channel design by integrating the advantages of the leaf vein-shaped and lung-shaped channels. A 3D multi-physics fuel cell model is used to verify the feasibility and superiority of the bionic snowflake design in improving fuel cell performance and reducing the pumping work. The local pressure distribution, oxygen distribution, water distribution, and current density distribution are used to reveal the enhancement mechanism of the new snowflake flow channel. The multi-objective and multi-parameter genetic algorithm optimization further improves the flow uniformity. The main conclusions are as follows.

(1) In overall performance, particularly in the concentration polarization, the snowflake flow channel fuel cell is better than the leaf flow channel, the lung flow channel, and the dual serpentine flow channel. For the peak power density, the snowflake channel design has a 27% improvement over the leaf vein channel, 66% improvement over the lung channel, and 45.5% improvement over the dual serpentine channel.

(2) The snowflake bionic flow channel has significant superiority in reducing pressure drop punishment for the fuel cell. Such superiority can fully diffuse the reaction gas, resulting in a uniform oxygen distribution in the entire flow channel, the diffusion layer. Moreover, the concentration polarization region of the entire fuel cell is delayed. The average current density in the membrane cross section is 27.86% larger than the dual serpentine flow channel, 13.58% larger than the leaf flow channel, and 135.14% larger than the lung flow channel.

(3) With a MOGA optimization, the snowflake shape-improved flow field is optimized to improve the uniformity of the entire flow field. The degree of deviation of the mass flow of each outlet before and after optimization was reduced by 29.98%.

## Figures and Tables

**Table 1:** Summary of flow channel design for PEM fuel cells.

Year	Peak Power Density/w cm^−2^	Pressure Drop/pa	Advantages	Disadvantages	Figure
2010 [5]	0.276	212	Small pressure drop.	The low channel flow rate results in low differential pressure across the battery. The diffusion ability of reactant to diffusion layer and drainage ability is poor, forming slug.	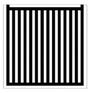
2010 [5]	0.45	3295	The large pressure drop between adjacent flow channels leads to the additional benefits of sub-rib convection bypass, which is beneficial to drainage and diffusion is the main transport mechanism of a single serpentine channel.	The parasitic loss and the pressure difference between the cathode and the anode are large and the membrane may be damaged. Increasing the cell area may cause an excessive pressure drop in the flow field.	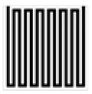
2010 [5]	0.423	5688	Despite its high water content, it does not affect battery performance. It is a promising form of the flow channel and convection under the ribs has proven to be very beneficial for battery performance.	Large pressure drop and uneven distribution.	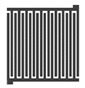
2012 [11]	0.55	250	Enhance the lateral transport phenomenon and increase the heat and mass transfer significantly. The inlet and outlet channels are adjacent and the pressure drop forces the reactant gas in the channel to flow through the gas diffusion layer and the catalyst layer and complementing the lack of reactants in the outlet channel. The outlet channel is smaller in size than the inlet, helping to remove moisture from the porous layer.	Around the circular area connecting the inlet and the outlet, especially where there is no flow channel, water will accumulate on the catalytic layer, reducing the contact area of the reaction.	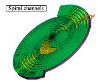
2013 [14]	0.207	small	Widely used flow field configuration with the advantage of reduced pressure.	There are disadvantages of uneven flow distribution and stagnation zone.	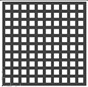
2013 [14]	0.587	N/A	Easy to manufacture, the shape of the channel takes into account the consumption of reactants. Speed distribution is uniform. Water management is good, management is good.		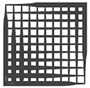
2012 [17]	1.1 (Ignore the effects of water)		As the reactants approach the outlet, the cross-sectional area of the main channel decreases and increased pressure forces the species to flow laterally, which will allow the reactants to spread more evenly across the diffusion layer.	There are many reactants attached to the outlet wall; there may be insufficient reactants between the downstream ribs.	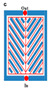
2014 [18]	0.57	6485	Using Murray’s law to narrow the third branch, the mass fraction of oxygen and oxygen is more uniform.	The designed inlet and outlet channels are disconnected and a large pressure drop is expected, which means that there is forced convection in the channel.	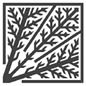
2015 [19]	0.302	29,900	More uniform reactant concentration and velocity distribution. Higher consumption of reactants. Geometry crosses each other to enhance the drainage effect of sub-rib flow. In relatively small channels, surface tension, inertial and viscous forces dominate, while gravity is negligible.	The designed inlet and outlet channels are disconnected and a large pressure drop is expected, which means that there is forced convection in the channel.	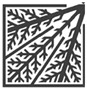
2017 [21]	0.088 (Methanol)		Good quality transportation ability, strong water management ability.	The overall effect is not ideal.	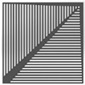
2017 [21]	0.088 (Methanol)		Good quality transportation ability, strong water management ability.	The overall effect is not ideal.	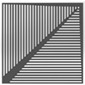
2018 [22]	0.54	2000	The repeated branching and fractal structure ensure a uniform distribution of oxygen within a given volume. The pressure drop is the same for each branch. The self-similar structure of the lung remains thermodynamically optimal.	The complex manufacturing process has an excellent performance in low humidity conditions; in contrast, it is easy to accumulate water.	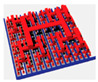
2019 [26]	0.83	5500	Enhances lateral flow of reactants and replenishment of fresh oxygen. Enhance water management. Membrane with good water content and better proton conductivity.	Large pressure drop, there will still be poor water management downstream.	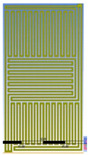
2015 [27]	0.44	4155	Improved standard finger-fork shape, reduced pressure drop, better uniformity.	Large pressure drop, the poor overall effect.	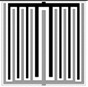
2015 [27]	0.42	208	Apply Murray’s Law to calculate branch width.	The reactants are unevenly distributed, the pressure drop is large, and the overall effect is worse than that of a single snake-type flow field.	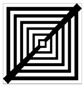
2017 [28]	1.6 (Output power at each pump work)		Efficiently partition reactants and remove products with minimal flow loss. Low pump power and low humidity.	Lower current density results in more severe membrane dehydration, which reduces performance.	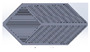
2017 [29]	0.99	3.17	Suitable fluid flow rate, no dead point. Reduced production costs and production time.	Uneven fuel distribution and low fuel utilization.	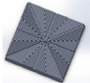

**Table 2:** Fuel cell parameters.

Parameters	Value
Anode reference current density, A m^−2^	10,000
Cathode reference current density, A m^−2^	20
Anode reference concentration, kmol m^−3^	1.0
Cathode reference concentration, kmol m^−3^	1.0
Anode concentration exponent	0.5
Cathode concentration exponent	1.0
Anode exchange coefficient	2
Cathode exchange coefficient	2
Open-circuit voltage, V	1.1
Current collector effective conductivity, 1 (Ω·m)^−1^	1 × 10^6^
Diffusion layer porosity	0.5
Diffusion layer viscous resistance, 1 m^−2^	1 × 10^12^
Diffusion layer effective conductivity, 1 (Ω·m)^−1^	5000
Catalyst layer porosity	0.5
Diffusion layer viscous resistance, 1 m^−2^	1 × 10^12^
Catalyst layer effective conductivity, 1 (Ω·m)^−1^	5000
Catalyst layer specific surface area, 1 m^−1^	2 × 10^5^
Reference diffusivity, m^2^ s^−1^	3 × 10^−5^
Membrane equivalent weight, kg kmol^−1^	1 100
Membrane protonic conduction coefficient	1.0
Membrane protonic conduction exponent	1.0
Membrane effective conductivity, 1 (Ω·m)^−1^	1 × 10^−16^

**Table 3:** The pressure drops for the PEM fuel cells.

Voltage	Pressure Drop			
(V)	Dual Serpentine	Leaf Shape	Lung Shape	Snowflake Shape
	Range (pa)	Range (pa)	Range (pa)	Range (pa)
0.2	0.21–59.23	8.59–17.65	1.41–14.11	0.09–1.14
0.3	0.21–59.49	8.06–16.55	1.40–13.80	0.09–1.15
0.4	0.21–59.56	8.64–17.34	1.40–14.17	0.09–1.16
0.5	0.22–60.02	8.37–17.00	1.37–10.82	0.09–1.09
0.6	0.22–59.81	8.30–16.83	1.30–9.80	0.09–1.17
0.7	0.23–59.91	8.19–16.57	1.27–9.66	0.09–1.16
0.8	0.22–58.44	7.81–16.08	1.25–5.55	0.08–1.10
0.9	0.22–54.82	7.78–15.88	1.21–1.59	0.08–1.06
1.0	0.22–54.28	7.85–15.97	4.77–7.16	0.08–1.04

**Table 4:** Multi-objective genetic algorithm parameters.

Input			Output		
	Before Optimization	After Optimization		Before Optimization	After Optimization
P1	4.5 mm	4.1128	P19 _main_	1.257 × 10^−7^	1.1706 × 10^−7^
P2	4.5 mm	4.3572	P20 _branch_	1.0541 × 10^−7^	9.8605 × 10^−8^
P3	4.5 mm	5.1785	P21 _branch_	1.0624 × 10^−7^	1.1115 × 10^−7^
P4	4.5 mm	5.2118	P22 _main_	8.9896 × 10^−8^	1.0124 × 10^−7^
P5	4.5 mm	5.2723	P23 _branch_	1.6012 × 10^−7^	1.6043 × 10^−7^
P6	4.5 mm	5.293	P24 _main_	9.105 × 10^−7^	1.0011 × 10^−7^
P7	4.5 mm	4.5752	P25 _main_	1.2435 × 10^−7^	1.0865 × 10^−7^
P8	4.5 mm	5.2457	P26 _branch_	1.0516 × 10^−7^	1.1415 × 10^−7^
P9	4.5 mm	3.7166	P27 _branch_	1.513 × 10^−7^	9.6089 × 10^−8^
P10	4.5 mm	5.2221	P28 _main_	8.8445 × 10^−8^	9.4439 × 10^−8^
P11	4.5 mm	5.2697	p29 _branch_	1.5974 × 10^−7^	1.5435 × 10^−7^
P12	4.5 mm	3.8161	P30 _main_	8.9976 × 10^−7^	9.4862 × 10^−8^
P13	4.5 mm	5.1571	P31_deviation_	2.1188 × 10^−14^	1.4835 × 10^−14^
P14	4.5 mm	4.2994			
P15	4.5 mm	3.7291			
P16	4.5 mm	3.819			
P17	4.5 mm	5.2364			
P18	4.5 mm	4.7414			

## Data Availability

Not applicable.

## Nomenclature

**Par.**	**Definitions**	**Par.**	**Definitions**
P_op_	Operating pressure, Pa	*ε*	Porosity
R	Universal gas constant, 8.314 J mol^−1^ K^−1^	*ρ*	Density, kg m^−3^
*T*	Operating temperature, K	*u*	Velocity vector, m s^−1^
X_k_	Mole fraction of substance	*μ*	Viscosity, kg m^−1^ s^−1^
*C*_k_	Mass fraction of substance	*ƞ*	Overpotential
*C*_F_	Quadratic drag factor	*λ*	Water content in membrane
*k*_p_	Permeability, m^2^	*ρ*_dry_	Membrane dry density, kg m^−3^
*k*_rg_	Relative permeability of the gaseous mixture	*σ*_s_	Electron conductivity, s m^−1^
*D*_k,eff_	Effective diffusion coefficient of substance k	*σ*_m_	Proton conductivity, s m^−1^
F	Faraday constant, 96.487 °C mol^−1^	*Φ*s	Electronic phase potential, V
*j*	Transfer current density, A m^−3^	*Φ*_m_	Ionic phase potential, V
*M*	Molecular weight, kg mol^−1^	*n*_d_	Electro-osmotic drag coefficient
*i*_m_	Current density in the membrane, A m^−2^	*P*_c_	Capillary pressure, Pa
*M*_m_	Membrane equivalent weight, kg mol^−1^	Subscripts	
*D*_λ_	Water diffusivity in the membrane	a	Anode
*A*_j0_	Exchange current density, A m^−3^	c	Cathode
*C*	Mass fraction	l	Liquid phase
*V*_cell_	Operating voltage v	ref	Reference value
